# Assessment of aromatic amides in printed food contact materials: analysis of potential cleavage to primary aromatic amines during simulated passage through the gastrointestinal tract

**DOI:** 10.1007/s00204-022-03254-w

**Published:** 2022-03-05

**Authors:** Nataly Bittner, Andy Boon, Evert H. Delbanco, Christof Walter, Angela Mally

**Affiliations:** 1 Department of Toxicology, University of Würzburg, Versbacher Str. 9, 97078 Würzburg, Germany; 2 Sun Chemical, Sargasso Building, Five Arches Business Centre, Maidstone Road, Sidcup, UK; 3 Siegwerk Druckfarben AG & Co. KGaA, Alfred-Keller-Straße 55, 53721 Siegburg, Germany; 4 Verband der deutschen Lack- und Druckfarbenindustrie e.V. (VdL), Mainzer Landstraße 55, 60329 Frankfurt, Germany

**Keywords:** Aromatic amides, Primary aromatic amine, Food contact materials, Simulated digestion

## Abstract

Recent analyses conducted by German official food control reported detection of the aromatic amides *N*-(2,4-dimethylphenyl)acetamide (NDPA), *N*-acetoacetyl-m-xylidine (NAAX) and 3-hydroxy-2-naphthanilide (Naphthol AS) in cold water extracts from certain food contact materials made from paper or cardboard, including paper straws, paper napkins, and cupcake liners. Because aromatic amides may be cleaved to potentially genotoxic primary amines upon oral intake, these findings raise concern that transfer of NDPA, NAAX and Naphthol AS from food contact materials into food may present a risk to human health. The aim of the present work was to assess the stability of NDPA, NAAX and Naphthol AS and potential cleavage to 2,4-dimethylaniline (2,4-DMA) and aniline during simulated passage through the gastrointestinal tract using static in vitro digestion models. Using the digestion model established by the National Institute for Public Health and the Environment (RIVM, Bilthoven, NL) and a protocol recommended by the European Food Safety Authority, potential hydrolysis of the aromatic amides to the respective aromatic amines was assessed by LC–MS/MS following incubation of the aromatic amides with digestive fluid simulants. Time-dependent hydrolysis of NDPA and NAAX resulting in formation of the primary aromatic amine 2,4-DMA was consistently observed in both models. The highest rate of cleavage of NDPA and NAAX was recorded following 4 h incubation with 0.07 M HCl as gastric-juice simulant, and amounted to 0.21% and 0.053%, respectively. Incubation of Naphthol AS with digestive fluid simulants did not give rise to an increase in the concentration of aniline above the background that resulted from the presence of aniline as an impurity of the test compound. Considering the lack of evidence for aniline formation from Naphthol AS and the extremely low rate of hydrolysis of the amide bonds of NDPA and NAAX during simulated passage through the gastrointestinal tract that gives rise to only very minor amounts of the potentially mutagenic and/or carcinogenic aromatic amine 2,4-DMA, risk assessment based on assumption of 100% cleavage to the primary aromatic amines would appear to overestimate health risks related to the presence of aromatic amides in food contact materials.

**Supplementary Information:**

The online version contains supplementary material available at 10.1007/s00204-022-03254-w.

## Introduction

Azo pigments are widely used as colourants in various consumer products, including printed food contact materials. According to regulations issued by The Swiss Federal Department of Home Affairs (FDHA, Annex 10 of the Ordinance of the FDHA on materials and articles intended to come into contact with foodstuffs) (FDHA 2020) and/or The German Federal Ministry of Food and Agriculture (Twenty-First Ordinance amending the German Consumer Goods Ordinance) (BMEL 2016), several azo pigments such as Pigment Yellow 174 (CAS 78952-72-4), Pigment Yellow 188 (CAS 23792-68-9) and Pigment Red 268 (CAS 16403-84-2) are permitted to be used for printing of food contact materials provided that a detectable transfer of the pigment into foods (defined as 0.01 mg/kg food) does not occur. While these regulations serve to ensure food safety by limiting any potential transfer of the azo pigments to foods, recent data suggest that aromatic amides, which are chemical substructures of these colourants and are likely to be present either as impurities or degradation products, may migrate into foods. In particular, recent analyses conducted by German official food control reported detection of the aromatic amides *N*-(2,4-dimethylphenyl)acetamide (NDPA), *N*-acetoacetyl-m-xylidine (NAAX) and 3-hydroxy-2-naphthanilide (Naphthol AS) in cold water extracts from certain food contact materials made from paper or cardboard, including paper straws, paper napkins, and cupcake liners (BfR 2019). These findings raise concern that transfer of NDPA, NAAX and Naphthol AS from food contact materials into food may present a risk to human health.

To date, there is no or at best very limited information on the toxicity of NDPA, NAAX and Naphthol AS. Acute oral toxicity of the three acid amides is low. In mice, the oral LD_50_ of NDPA was 1300 mg/kg body weight (bw) (Starmer et al. 1971), whereas oral LD_50s_ of NAAX and Naphthol AS in rats were reported to be 1995 mg/kg bw (ECHA 2021a) and > 5 g/kg bw (Kakada et al. 1986), respectively. In a repeated dose 28-day oral rodent toxicity study on NAAX conducted according to OECD 407, male and female rats (*n* = 5/group) were administered NAAX at 0, 8, 40, 200 mg/kg bw/day (ECHA 2021a). Treatment-related effects were observed in the highest dose groups of both sex, and included decreased red blood cell count, hemoglobin, and hematocrit in males, increased absolute and relative liver weight, and pale kidneys in males and females (ECHA 2021a). Neoplastic or non-neoplastic histopathological changes were not observed. Based on the effects observed at 200 mg/kg bw, a no-observed-adverse-effect-level (NOAEL) of 40 mg/kg bw was established. NAAX showed no mutagenic activity in *S. typhimurium* TA 1535, TA 1537, TA1538, TA 98, TA 100, and E. coli WP2 uvr A with and without metabolic activation (ECHA 2021a).

A combined repeated dose toxicity study with the reproduction/developmental toxicity screening test conducted according to OECD 422 reported no effects of Naphthol AS on parents and offspring F1, resulting in a no-observed-effect-level (NOEL) of 1000 mg/kg bw/day for Naphthol AS (JCHECK 2021). Naphthol AS was reported to be negative in an in vitro mammalian chromosomal aberration test and in *S. typhimurium* TA 1535, TA 1537, TA1538, TA 98, TA 100, and *E. coli* WP2 uvr A with and without metabolic activation (JECDB 2021). There are no data on repeated dose toxicity or genotoxicity of NDPA in mammalian cells. A recent bacterial mutation assay in *S. typhimurium* TA 1535, TA 1537, TA 98, TA 100, and *E. coli* WP2 uvr A conducted in compliance with OECD 471 and Council Regulation (EC) No. 440/2008 (Test method B.13/14) provided no evidence for mutagenicity of NDPA in the absence and presence of S9 metabolic activation using both plate incorporation and pre-incubation protocols (ERBC 2020).^1^

**Fig. 1:**
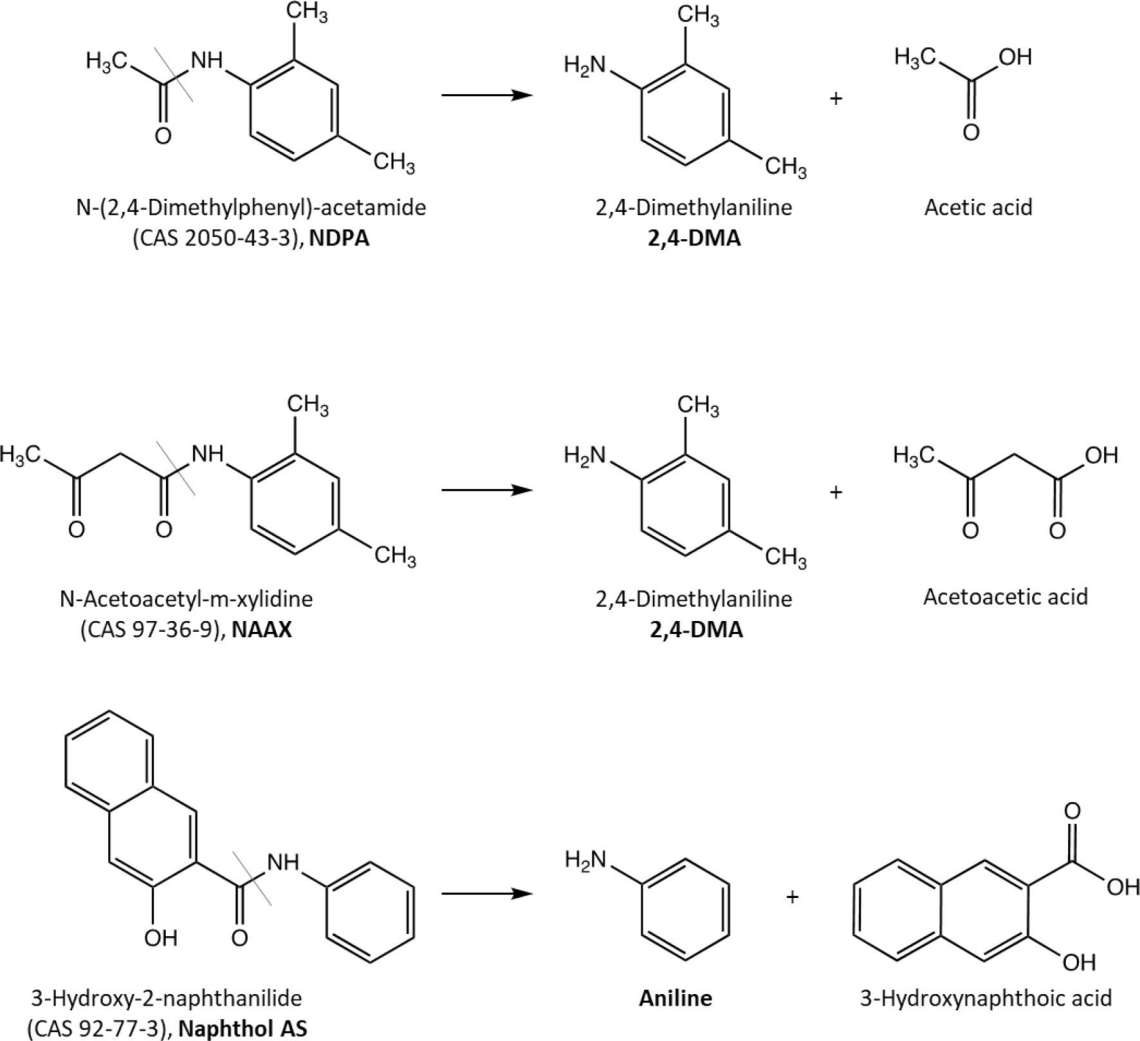
Chemical structures of *N*-(2,4-dimethylphenyl)acetamide (NDPA), *N*-acetoacetyl-m-xylidine (NAAX) and 3-hydroxy-2-naphthanilide (Naphthol AS) and cleavage to the primary aromatic amines 2,4- dimethylaniline (2,4-DMA) and aniline

However, there is concern that upon oral intake, NDPA, NAAX and Naphthol AS may be cleaved to potentially mutagenic and/or carcinogenic aromatic amines, i.e., 2,4- dimethylaniline (2,4-DMA) in the case of NDPA and NAAX and aniline in the case of Naphthol AS (Fig. 1). While aniline was consistently negative in the Ames test (De Flora 1981; Haworth et al. 1983; Przybojewska 1999), it was found to induce chromosomal effects in cultured mammalian cells at high, millimolar concentrations (ECHA 2021b; Galloway et al. 1987; Ishidate et al. 1988). In vivo, micronuclei and/or chromosomal aberrations were observed in the bone marrow of rats (Bomhard 2003) and mice (Ashby et al. 1991; Westmoreland and Gatehouse 1991), albeit at high doses associated with hematotoxicity (ECHA 2021b). The clastogenic effects are considered to be caused by an indirect mechanism linked to erythrotoxicity of aniline (ECHA 2021b). The Scientific Committee on Occupational Exposure Limits (SCOE) therefore considered the in vivo genotoxic potential of aniline to be low (Bolt et al. 2016). Data on genotoxicity of 2,4-DMA are somewhat inconsistent. Positive results were obtained for mutagenicity in *S. typhimurium* TA100 in the presence of metabolic activation (Chung et al. 1981; Kimmel et al. 1986; Nohmi et al. 1983; Zeiger et al. 1988) and for unscheduled DNA synthesis in rat hepatocytes (Williams et al. 1989; Yoshimi et al. 1988). In vivo, DNA-strand breaks were observed in bone marrow and liver of mice treated with 2,4-DMA (Przybojewska 1997, 1999). In a recent study, 2,4-DMA was shown to induce γ-H2AX formation in cultured human urothelial and hepatic cells at millimolar concentrations (Qi et al. 2018). DNA damage induced by 2,4-DMA was associated with reactive oxygen formation and could be blocked by a specific CYP2E1 inhibitor, leading the authors to conclude that reactive oxygen species produced by CYP2E1-mediated metabolism of 2,4-DMA play an important role (Qi et al. 2018).

In light of the genotoxic and potentially carcinogenic effects of aniline and 2,4-DMA, the aim of the present work was to assess the stability of NDPA, NAAX and Naphthol AS and potential cleavage to 2,4-DMA and aniline during simulated passage through the gastrointestinal tract using static in vitro digestion models. To date, there is no generally agreed standardized method to simulate digestion in the gastrointestinal tract in vitro. A number of static in vitro digestion models have been used to assess liberation and stability of drugs from drug formulations (Asafu-Adjaye et al. 2007; Stippler et al. 2004) or natural food constituents and contaminants from food (Dall'Erta et al. 2013; Islam et al. 2014; Rha et al. 2019; Versantvoort et al. 2005), consumer products (Brandon et al. 2006) and soil samples (Oomen et al. 2003) during passage through the gastrointestinal tract (Punt et al. 2017). These models differ slightly in the complexity and composition of simulated digestive fluids and incubation times (Brodkorb et al. 2019). In addition, in its recent Note for Guidance for the safety assessment of substances to be used in plastic food contact materials, the European Food Safety Authority (EFSA) provided a protocol for assessing hydrolysis of monomers and other additives to plastic food contact materials in digestive fluid simulants (EFSA 2008).

The present investigation thus utilized the digestion model established by the National Institute for Public Health and the Environment (RIVM, Bilthoven, NL) (Oomen et al. 2003; Versantvoort et al. 2005) as well as the protocol recommended by EFSA (EFSA 2008) to determine the rate of hydrolysis of NDPA, NAAX and Naphthol AS during simulated passage through the gastrointestinal tract.

## Materials and methods

### Chemicals and reagents

2,4-Dimethylaniline (2,4-DMA) (CAS No. 95-68-1; 99.4% purity), aniline (CAS No. 62–53-3; > 99.5% purity), 2’,4’-Dimethylacetanilide (NDPA) (CAS No. 2050-43-3; > 98% purity) and 2’,4’-Dimethylacetoacetanilide (NAAX) (CAS No. 97-36-9; > 99% purity) were acquired from Merck (Darmstadt, Germany). 2-Naphthalenecarboxamide (Naphthol AS) (CAS No. 92-77-3; > 99% purity) was acquired from Fisher Scientific (Leicestershire, UK). Unless otherwise indicated, all other chemicals were from Merck (Darmstadt, Germany), Sigma-Aldrich (Taufkirchen, Germany), AppliChem (Darmstadt, Germany), Grüssing (Filsum, Germany) or Roth (Karlsruhe, Germany).

### Purity assessment of NDPA, NAAX and Naphthol AS

While NDPA, NAAX and Naphthol AS were obtained of highest purity available, the substances were subjected to LC–MS/MS analysis to assess potential contamination with 2,4-DMA or aniline prior to in vitro digestion. To this end, NDPA and NAAX were each dissolved in H_2_O at a concentration of 318 nmol/mL. Due to its lower solubility in aqueous solutions, Naphthol AS was dissolved in H_2_O at a concentration of 43.7 nmol/mL. Of each solution, 10 µL were injected into the LC–MS/MS system and analyzed by monitoring *m/z* transitions characteristic for each test compound, i.e., NDPA *(m/z 164.1 → 122.1)*, NAAX (*m/z 206.1 → 122.1*) and Naphthol AS (*m/z 264.1 → 171.1*), and the potential cleavage products 2,4-dimethylaniline (2,4-DMA) (*m/z 122.0 → 107.1*) and aniline (*m/z 94.1 → 77.1*) using the method described below.

### In vitro digestion based on EFSA note for guidance for food contact materials

Incubations of test compounds were performed in digestive fluid simulants prepared according to the protocol provided in the EFSA Note for Guidance for Food Contact Materials (EFSA 2008). The composition of saliva simulant, gastric-juice simulant and intestinal-fluid simulant is shown in Table 1. NDPA and NAAX were each incubated in saliva simulant (37 °C; 0.5 h), gastric-juice simulant (37 °C; 1, 2 and 4 h) and intestinal-fluid simulant (37 °C; 1, 2 and 4 h) at a final concentration of 318 nmol/mL, while the final concentration of Naphthol AS in each digestive fluid simulant was 43.7 nmol/mL due to its lower solubility in aqueous solutions. Samples were immediately frozen and kept at − 20 °C until analysis. For analysis of cleavage products via LC–MS/MS, samples were thawed one at a time, centrifuged, and immediately injected into the LC–MS system. For analysis of NDPA, NAAX and Naphthol AS, an aliquot (10 µL) of each sample was diluted 1:100 with H_2_O and injected into the LC–MS/MS system. Each experiment was conducted in triplicate.

**Table 1:** Composition of digestive fluid simulants based on EFSA Note for Guidance for Food Contact Materials (EFSA 2008)

Saliva simulant	Gastric-juice simulant	Intestinal-fluid simulant
4.2 g NaHCO_3_	0.07 M HCl	6.8 g KH_2_PO_4_
0.5 g NaCl		190 ml 0.2 N NaOH
0.2 g K_2_CO_3_		10 g Pancreatin
H_2_O ad 1L		0.5 g Na-Taurocholate
		H_2_O ad 1L
pH 9	pH 1.2 ± 0.1	pH 7.5 ± 0.1

### In vitro digestion based on the model of Versantvoort et al. (2005)

To mimic the digestive process in the oral cavity, stomach, and small intestine, the in vitro digestion procedure based on Versantvoort et al. (2005) involves sequential incubation of the test material with simulated saliva (pH 6.8, 37 °C, 5 min), gastric juice (pH 2–3, 37 °C, 2 h) and intestinal juice/bile (pH 6.5–7, 37 °C, 2 h) and removal of aliquots for analysis after each incubation step (Dall'Erta et al. 2013; Islam et al. 2014; Oomen et al. 2003; Rha et al. 2019). This procedure was slightly modified to increase the maximum incubation times to 0.5 h in saliva and 4 h in gastric juice and intestinal-fluid simulant as proposed by EFSA (2008) and to investigate the kinetics of hydrolysis in gastric and intestinal juice. Digestive simulants (Table 2) were prepared as described in Versantvoort et al. (2005) except that mucin (saliva, gastric juice) and lipase (intestinal fluid) were omitted as they were not commercially available at the time. Stock solutions of NDPA (5 mg/mL in H_2_O/acetonitrile 50:50), NAAX (2 mg/mL in ethanol) and Naphthol AS (1 mg/mL in ethanol) were prepared and further diluted in H_2_O as appropriate to achieve a final concentration of test compound of 318 nmol/mL for NDPA and NAAX and 43.7 nmol/mL for Naphthol AS in each incubation mixture. To 100 µL solution of test compound, 120 µL saliva simulant (preincubated for 0.5 h at 37 °C) were added and the mixture was incubated at 37 °C for 0.5 h with constant shaking to test for hydrolysis in saliva. To assess cleavage of NDPA and NAAX during simulated passage through the oral cavity and stomach, 100 µL solution of test compound was first incubated with 120 µL saliva simulant for 0.5 h; 240 µL gastric-juice simulant was then added and the mixture was incubated at 37 °C for 1, 2 and 4 h with constant shaking. For analysis of cleavage during simulated passage through the entire gastrointestinal tract, 100 µL were first incubated with 120 µL saliva for 0.5 h; the mixture was subsequently incubated with 240 µL gastric-juice simulant for 2 h, before addition of 240 µL intestinal juice, 120 µL bile and 40 µL NaHCO_3_ and incubation at 37 °C for 1, 2 and 4 h with constant shaking. Samples were immediately frozen and kept at − 20 °C until analysis. For analysis of cleavage products via LC–MS/MS, samples were thawed one at a time, centrifuged and immediately injected into the LC–MS/MS system. For analysis of NDPA, NAAX and Naphthol AS, an aliquot (10 µL) of each sample was diluted 1:100 with H_2_O and injected into the LC–MS/MS system. Each experiment was conducted in triplicate.

**Table 2:** Composition of digestive fluid simulants based on (Versantvoort et al. 2005)

	Saliva	Gastric juice	Duodenal juice	Bile juice
Inorganic solution	1.0 mL KCl 89.6 g/L	1.57 mL NaCl 175.3 g/L	4.0 mL NaCl 175.3 g/L	3.0 mL NaCl 175.3
	1.0 mL KSCN 20 g/L	300 µL NaH_2_PO_4_ 88.8 g/L	4.0 mL NaHCO_3_ 84.7 g/L	6.83 mL NaHCO_3_ 84.7 g/L
	1.0 mL NaH_2_PO_4_ 88.8 g/L 1.0 mL Na_2_SO_4_ 57 g/L 170 µL NaCl 175.3 g/L 2.0 mL NaHCO_3_ 84.7 g/L	920 µL KCl 89.6 g/L 1.8 mL CaCl_2_ 2 H_2_O 22.2 g/L 1.0 mL NH_4_Cl 30.6 g/L 650 µL HCl 37% g/g	1.0 mL KH_2_PO_4_ 8 g/L 630 µL KCl 89.6 g/L 1.0 mL MgCl_2_ 5 g/L 18 μL HCl 37% g/g	420 µL KCl 89.6 g/L 15 μL HCl 37% g/g
Organic solution	800 µL urea 25 g/L	1 mL glucose 65 g/L	400 µL urea 25 g/L	1.0 mL urea 25 g/L
		1 mL glucuronic acid 2 g/L		
		340 µL urea 25 g/L		
		1 mL glucosamine-HCl 33 g/L		
Further constituents	29 mg alpha-Amylase	100 mg BSA	900 µL CaCl_2_. 2 H_2_O 22.2 g/L	1.0 mL/L CaCl_2_ 2 H_2_O 22.2 g/L
	1.5 mg uric acid	250 mg pepsin	100 mg BSA	180 mg BSA
	2.5 mg mucin*	300 mg mucin*	900 mg pancreatin	3.0 g bile
pH	6.8 ± 0.2	1.30 ± 0.02	150 mg lipase* 8.1 ± 0.2	8.2 ± 0.2

Solutions of inorganic and organic constituents were augmented to 50 mL with distilled water. The inorganic and organic solutions were mixed, and further constituents were added and dissolved. The pH of the solutions was adjusted as indicated

*Note that mucin and lipase were not commercially available at the time and were therefore omitted

### LC–MS analysis

LC–MS/MS analyses were performed on an Agilent 1100 series LC coupled to an API 2000/Q-Trap mass spectrometer (Applied Biosystems/MDS Sciex, Concord, Canada). Samples were injected into the LC–MS/MS system through an Agilent 1100 series autosampler. Separations were carried out on a ReproSil-Pur C18-AQ column (2 mm × 150 mm, 3 µm; Dr. Maisch; Ammerbuch, Germany). Gradient elution of analytes was carried out with water + 0.1% formic acid (solvent A) and acetonitrile + 0.1% formic acid (solvent B). Initially, solvent A was held isocratic for 3 min at 97%, followed by a linear gradient to 90% B in 3 min. These conditions were held for further 9 min. Within 1 min, the gradient decreased linear to 3% B and remained at initial conditions until the end of analysis (25 min). A flow rate of 0.2 mL/min was used. For each run, 10 μL of the respective sample were injected by the autosampler.

The API 2000/Q-Trap mass spectrometer was operated with a Turbo Ion Spray source in the positive ion mode with a voltage of 4000 V. Spectral data were recorded with N_2_ as the heater gas at 450 °C and as the collision gas (CAD = Medium) in the multiple reaction monitoring mode (MRM). The analytes with retention times and *m/z* transitions monitored are summarized in Table 3. Matrix-matched calibration curves (6–8 points) were prepared from solutions of 2,4-DMA and aniline in each of the digestive fluid simulants. Calibration curves for the aromatic amides NDPA, NAAX and Naphthol AS were prepared by dilution of stock solutions in H_2_O instead of digestive fluid simulants to prevent cleavage.

**Table 3:** HPLC–ESI–MS/MS parameters for aromatic amides and their potential cleavage products

Analyte	*m/z* Transition	Retention time
Aniline	94.1 → 77.1	2.9
2,4-DMA	122.0 → 107.1	10.4
NDPA	164.1 → 122.1	11.3
NAAX	206.1 → 122.1	11.4
Naphthol AS	264.1 → 171.1	13.4

The limits of detection (LOD) and quantification (LOQ) were determined by spiking digestive fluid simulants in triplicate with reference standards and evaluated using the signal-to-noise (S/N) ratio of 3:1 and 10:1, respectively. The extent of hydrolysis of aromatic amides was determined by subtracting the concentration of 2,4-DMA or aniline present as impurity (*t* = 0) from the concentration of 2,4-DMA and aniline measured after incubation with digestive fluid simulants.

### HPLC–DAD analysis

HPLC analyses of incubations of Naphthol AS in digestive fluid simulants were performed using a Hewlett-Packard HP 1090 HPLC–DAD system. Analytes were separated on a ReproSil-Pur C18-AQ (150 × 4.6 mm, 5 µm; Dr. Maisch; Ammerbuch, Germany with a C18-AQ guard-column and detected at 237 nm. Analytes were separated by gradient elution with water + 0.1% formic acid (solvent A) and acetonitrile + 0.1% formic acid (solvent B). Initially, solvent A was held isocratic for 3 min at 97%, followed by a linear gradient to 90% B in 3 min. These conditions were held for further 9 min. Initial conditions were reconstituted within 1 min.

## Results

### Analytical method development and purity assessment

**Fig. 2:**
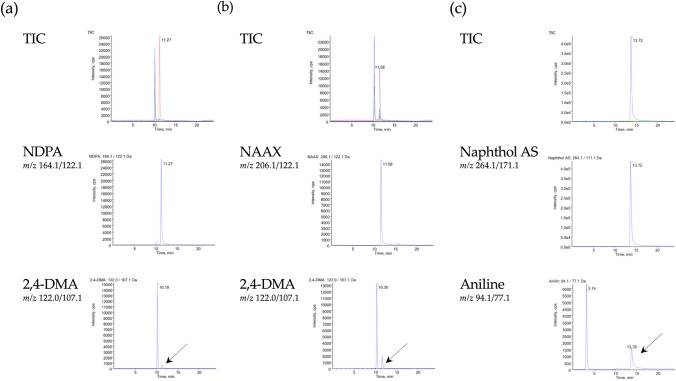
HPLC–ESI–MS/MS analysis of standard solutions containing **a** NDPA and 2,4-DMA (100 ng/mL each) **b** NAAX (400 ng/mL) and 2,4-DMA (100 ng/mL), and **c** Naphthol AS (18.4 µg/mL) and aniline (15.8 ng/mL) showing total ion chromatograms and mass transitions specific for each analyte. Importantly, analyses demonstrate chromatographic separation of each of the acid amides from their potential degradation product 2,4-DMA or aniline, as both NDPA and NAAX are degraded to 2,4-DMA during electrospray (**a** and **b** marked by arrow), while Naphthol AS gives rise to aniline during electrospray (**c** marked by arrow)

An LC–MS/MS-based analytical method was developed that allowed separation of the acid amides NDPA and NAAX from their potential degradation product 2,4-DMA (Fig. 2a, b) as well as separation of Naphthol AS from its potential degradation product aniline (Fig. 2c). Of note, all three acid amides were found to decompose to their respective degradation products during electrospray (Fig. 2), as evidenced by peaks with the same chromatographic retention time as NDPA, NAAX and Naphthol AS but with mass transitions corresponding to 2,4-DMA and aniline (indicated by arrows in Fig. 2). However, due to clear chromatographic separation of the analytes 2,4-DMA and aniline from NDPA, NAAX and Naphthol AS, artefactual formation during electrospray did not interfere with qualitative and quantitative analyses.

For quantitative analysis, standard solutions of 2,4-DMA and aniline were prepared both in solvent (acetonitrile; acetonitrile:H_2_O) and in the respective digestion fluids based on EFSA (2008) and Versantvoort et al. (2005) to account for matrix effects. Calibration was linear in the range of 1.0 ng/mL to 200 ng/mL for 2,4-DMA and in the range of 5–30 ng/mL for aniline. The limits of detection (LOD) and limits of quantification (LOQ) of 2,4-DMA and aniline were determined in each matrix (Table 4). The sensitivity of the method was found to decrease with increasing complexity of the matrix from saliva to gastric juice and duodenal juice based on Versantvoort et al. (2005).

**Table 4:** LOD and LOQ of 2,4-DMA and aniline in the digestive fluid simulants

	2,4-DMA		Aniline	
	LOD [ng/mL]	LOQ [ng/mL]	LOD [ng/mL]	LOQ [ng/mL]
EFSA (2008)				
Saliva	0.9	2.0	0.4	1.0
Gastric juice	0.4	1.0	0.9	2.6
Intestinal juice	1.0	3.3	2.3	7.5
Versantvoort et al. (2005)				
Saliva	0.3	1.0	0.5	1.2
Saliva + gastric juice	0.3	0.8	1.3	3.0
Saliva + gastric + intestinal Juice	0.7	1.5	0.7	1.7

**Fig. 3:**
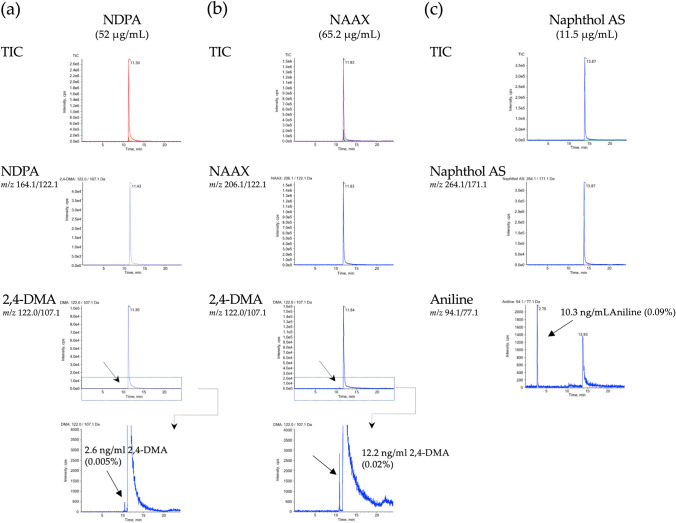
HPLC–ESI–MS/MS analysis of the test compounds **a** NDPA (318 nmol/mL = 52 µg/mL), **b** NAAX (318 nmol/mL = 65.2 µg/mL) and **c** Naphthol AS (43.7 nmol/mL = 11.5 µg/mL) showing total ion chromatograms and mass transitions specific for each analyte. Analyses demonstrate traces of the potential degradation product 2,4-DMA in solutions of NDPA (0.005%) and NAAX (0.02%) (**a**, **b**) as well as the presence of aniline as an impurity of Naphthol AS (0.09%). Note that both NDPA and NAAX are degraded to 2,4-DMA during electrospray, giving rise to signals with *m/z* transition 122.0/107.1 and identical retention times as the test compounds, whereas 2,4-DMA present as an impurity elutes at 10.16 min (see Fig. 2). Similarly, Naphthol AS forms aniline during electrospray, as evidenced by a signal with *m/z* transition 94.1/77.1 and same retention time as Naphthol AS. However, a further signal corresponding to aniline (retention time 3.12 min) is observed

Before investigating in vitro digestion of the acid amides, NDPA, NAAX and Naphthol AS were subjected to LC–MS/MS analysis to control for the presence of 2,4-DMA and aniline as potential impurities (Fig. 3). NDPA and NAAX were found to contain traces of 2,4-DMA, (0.005% and 0.02%, respectively), while Naphthol AS contained 0.09% aniline (Fig. 3).

### In vitro digestion of NDPA and NAAX based on EFSA note for guidance for food contact materials (2008)

**Fig. 4:**
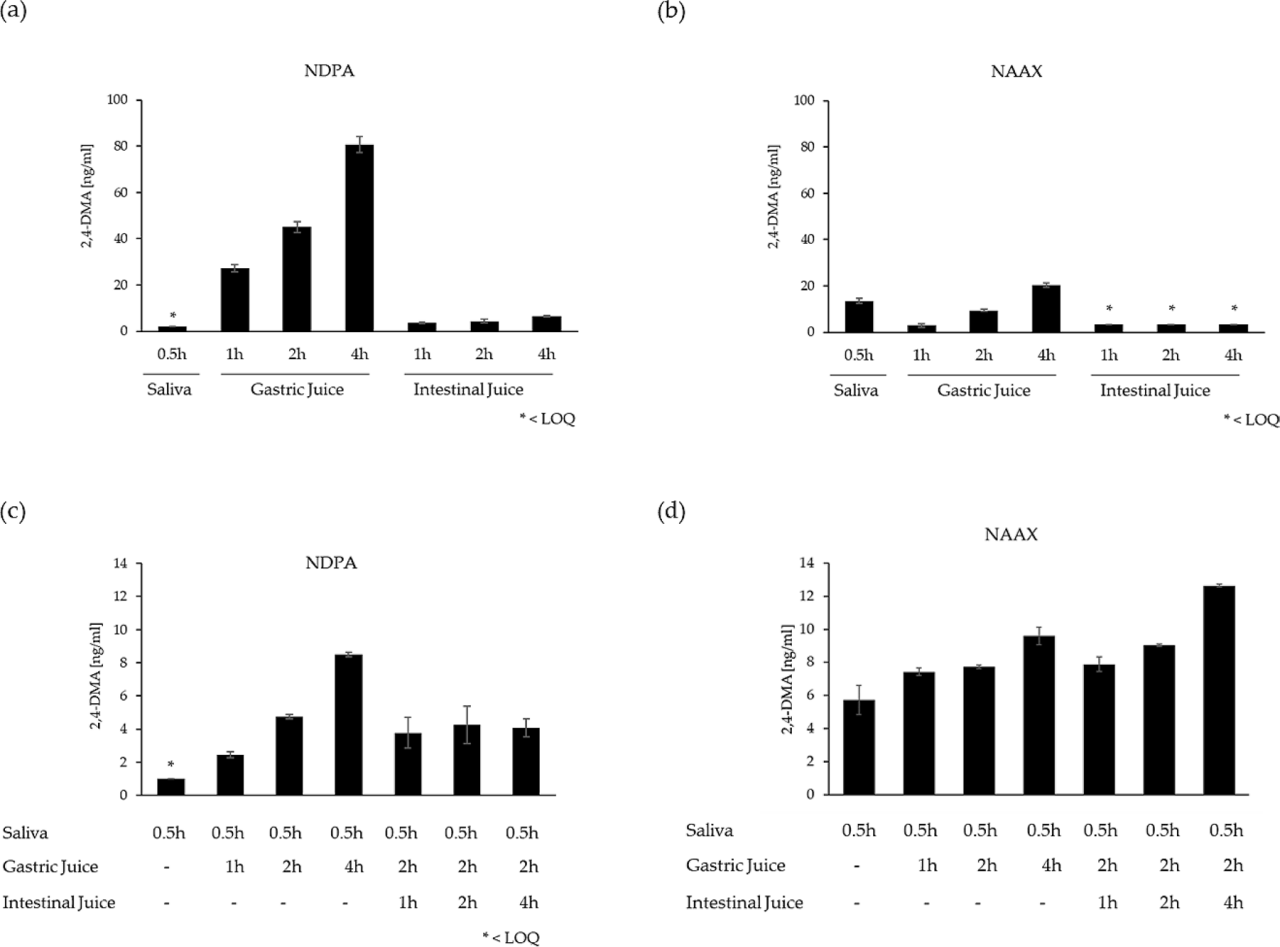
Quantitative analysis of cleavage of NDPA (**a**, **c**) and NAAX (**b**, **d**) to 2,4-DMA following in vitro digestion based on the protocol of EFSA (2008) (**a**, **b**) and Versantvoort et al. (2005) (**c**, **d**). The final concentration of test compound was 318 nmol/mL in each incubation. Data are presented as mean ± standard deviation (*n* = 3)

Digestion experiments of NDPA (318 nmol/mL) and NAAX (318 nmol/mL) were conducted using digestive fluid simulants according to EFSA (2008). In the EFSA digestion model, time-dependent hydrolysis of NDPA to 2,4-DMA was observed in 0.07 M HCL that served as gastric juice (1, 2 and 4 h), whereas no or only little cleavage (< 0.02%) was observed in saliva (0.5 h) and intestinal-fluid simulant (1, 2 and 4 h) (Fig. 4 and Supplemental Fig. 1). Quantitative analysis of 2,4-DMA in incubations of NDPA with gastric juice for 1, 2 and 4 h (*n* = 3) showed 0.07%, 0.12% and 0.21% hydrolysis of NDPA to 2,4-DMA, respectively (Table 5). Cleavage of NAAX to 2,4-DMA was evident in both saliva and gastric-juice simulant (Fig. 4 and Supplemental Fig. 1), whereas no hydrolysis was observed following incubation with intestinal-fluid simulant. Based on the determined concentration of 2,4-DMA in saliva and gastric-juice simulant, hydrolysis of NAAX to 2,4-DMA amounted to 0.035% in saliva, and 0.008%, 0.024 and 0.053% in gastric juice after 1, 2 and 4 h, respectively (*n* = 3). Quantitative analysis of NDPA and NAAX essentially confirmed the absence of substantial cleavage of the aromatic amides (Supplemental Fig. 3). However, due to analytical variability, monitoring substrate loss at such low rates of hydrolysis was not suitable to accurately quantify the extent of cleavage.

**Table 5:** Quantitative analysis of cleavage of NDPA (318 nmol/mL) and NAAX (318 nmol/mL) to 2,4-DMA following in vitro digestion based on the protocol of EFSA (2008) and Versantvoort et al. (2005)

Test compound	EFSA (2008)					Versantvoort et al. (2005)				
	Saliva	Gastric Juice	Intestinal Juice	2,4-DMA		Saliva	Gastric Juice	Intestinal Juice	2,4-DMA	
				(ng/mL)	[%]				(ng/mL)	[%]
NDPA	0.5 h	–	–	< 2.0*	< 0.005	0.5 h	–	–	< 0.1*	< 0.003
	–	1 h	–	27.3 ± 1.5	0.071 ± 0.004	0.5 h	1 h	–	2.5 ± 0.2	0.006 ± 0.001
	–	2 h	–	45.0 ± 2.7	0.117 ± 0.006	0.5 h	2 h	–	4.7 ± 0.2	0.012 ± 0.000
	–	4 h	–	80.8 ± 3.5	0.209 ± 0.009	0.5 h	4 h	–	8.5 ± 0.1	0.022 ± 0.000
	–	–	1 h	3.6 ± 0.4	0.009 ± 0.001	0.5 h	2 h	1 h	3.8 ± 0.9	0.010 ± 0.002
	–	–	2 h	4.4 ± 0.8	0.011 ± 0.002	0.5 h	2 h	2 h	4.3 ± 1.1	0.011 ± 0.003
	–	–	4 h	6.5 ± 0.4	0.017 ± 0.001	0.5 h	2 h	4 h	4.1 ± 0.5	0.011 ± 0.001
NAAX	0.5 h	–	–	13.5 ± 1.2	0.035 ± 0.003	0.5 h	–	–	5.7 ± 0.9	0.015 ± 0.002
	–	1 h	–	2.9 ± 0.8	0.008 ± 0.002	0.5 h	1 h	–	7.4 ± 0.2	0.019 ± 0.001
	–	2 h	–	9.4 ± 0.4	0.024 ± 0.001	0.5 h	2 h	–	7.7 ± 0.1	0.020 ± 0.000
	–	4 h	–	20.2 ± 0.9	0.053 ± 0.003	0.5 h	4 h	–	9.6 ± 0.5	0.025 ± 0.001
	–	–	1 h	< 3.3 *	< 0.009	0.5 h	2 h	1 h	7.9 ± 0.4	0.021 ± 0.001
	–	–	2 h	< 3.3 *	< 0.009	0.5 h	2 h	2 h	9.0 ± 0.1	0.024 ± 0.000
	–	–	4 h	< 3.3 *	< 0.009	0.5 h	2 h	4 h	12.6 ± 0.1	0.033 ± 0.000

Data are presented as concentration of 2,4-DMA (ng/mL) and percent cleavage of NDPA or NAAX (mean ± standard deviation, *n* = 3)

^*^LOQ

### In vitro digestion of NDPA and NAAX based on the model of Versantvoort et al. (2005)

Consistent with results obtained using the in vitro digestion model based on EFSA Note for Guidance for Food Contact Materials (EFSA 2008), hydrolysis of NDPA to 2,4-DMA was observed predominantly following incubation with gastric-juice simulant. In gastric juice, the concentration of 2,4-DMA formed by cleavage of NDPA increased with increasing incubation time from 0.006% after 1 h to 0.012 and 0.021% after 2 and 4 h, respectively (Fig. 4 and Supplemental Fig. 2; Table 5), whereas subsequent incubation in intestinal juice simulant for up to 4 h did not further increase the concentration of 2,4-DMA (Fig. 4; Table 5). Of note, the extent of cleavage of NDPA to 2,4-DMA following sequential incubation with saliva (pH 6.8) and gastric-juice simulants (pH 2–3) based on the model of Versantvoort et al. (2005) was almost an order of magnitude lower as compared to the protocol by EFSA (2008), which utilizes 0.07 M HCl (pH 1.2) as gastric-juice simulant.

In contrast to NDPA, hydrolysis of NAAX to 2,4-DMA was evident in all three digestive fluid simulants, i.e., in saliva, gastric juice and intestinal fluid, albeit at low rates. In gastric juice and intestinal-fluid simulant, the concentration of 2,4-DMA formed by cleavage of NAAX increased with increasing incubation time (Fig. 4 and Supplemental Fig. 2; Table 5). With 0.033%, the highest rate of hydrolysis was thus observed following subsequent incubation of NAAX with saliva (0.5 h), gastric juice (2 h) and intestinal-fluid simulant for 4 h (Table 5), whereby the extent of cleavage in saliva alone accounted for 0.015%. Similar to NDPA, a higher rate of hydrolysis of NAAX was observed in the EFSA (2008) fluid simulants as compared to the more complex digestive fluid simulants based on the model of Versantvoort et al. (2005). In line with results obtained with the EFSA (2008) model, quantitative analysis of NDPA and NAAX following sequential incubation with digestive fluid simulants confirmed the absence of substantial cleavage of the aromatic amides (Supplemental Fig. 3).

### In vitro digestion of Naphthol AS based on the models of EFSA (2008) and Versantvoort et al. (2005)

**Fig. 5:**
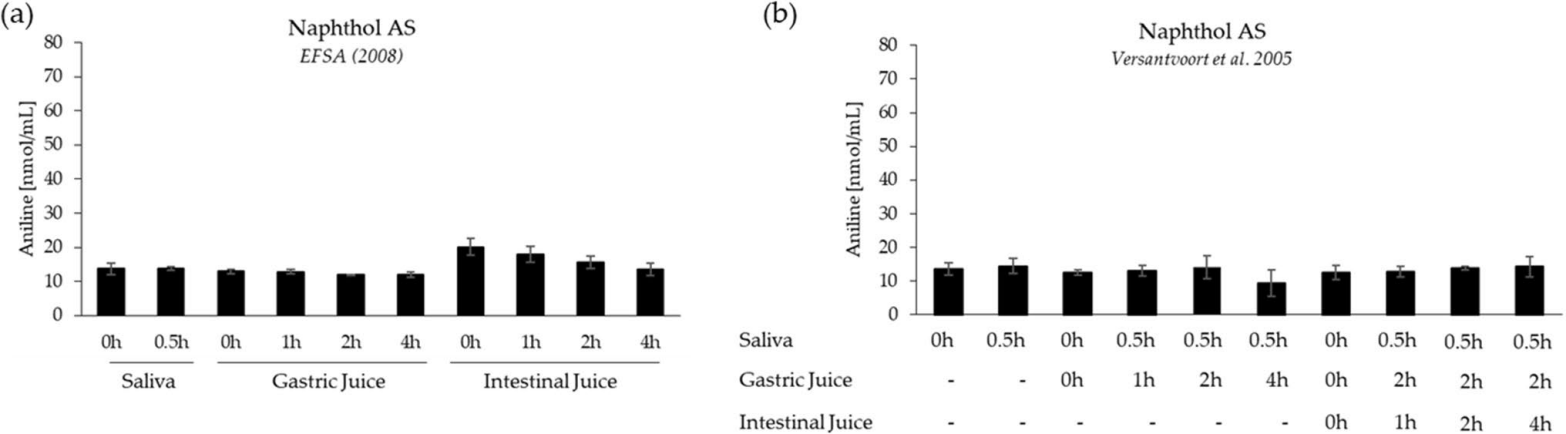
Quantitative analysis of potential cleavage of Naphthol AS to aniline following in vitro digestion based on the protocol of **a** EFSA (2008) and **b** Versantvoort et al. (2005). Data are presented as mean ± standard deviation (*n* = 3). The final concentration of test compound in each incubation mixture was 43.7 nmol/mL. To account for background contamination of Naphthol AS with aniline, additional samples (*t* = 0 h) were generated by addition of Naphthol AS to the respective digestive fluid, centrifugation and immediate injection into the LC–MS system

In contrast to the small but noticeable extent of cleavage of NDPA and NAAX to 2,4-DMA, incubation of Naphthol AS with digestive fluid simulants did not give rise to an increase in the concentration of aniline above the background that resulted from the presence of aniline as an impurity of the test compound (Fig. 5). Of note, however, the concentrations of Naphthol AS determined after incubation with gastric fluid simulant were significantly lower than the nominal concentrations added. This observation was confirmed by HPLC with UV detection (237 nm) (data not shown), indicating that this was not due to reduced ionization efficiency caused by matrix components. Importantly, however, no additional signals that would indicate degradation of Naphthol AS were detected. To understand the reason for the apparent substrate loss, Naphthol AS was spiked to the respective digestive fluid simulants and the solutions were immediately measured by LC–MS/MS. Recovery of Naphthol AS in the spiked gastric fluid simulants was 35% in the EFSA (2008) model and 55% in the model based on Versantvoort et al. (2005), and thus in the same range as after incubation in the respective fluid for up to 4 h (Supplementary Fig. 4). Importantly, however, complete recovery of Naphthol AS was observed after sequential incubation with saliva, gastric juice and intestinal juice, indicating that Naphthol AS was not degraded (Supplementary Fig. 4). Further experiments confirmed reduced solubility of Naphthol AS at low pH and dissolution of the precipitate after re-adjusting the pH to neutral conditions. While this issue precluded accurate quantification of substrate loss, these data combined with quantitative analysis of the presumed cleavage product aniline support the conclusion that Naphthol AS does not undergo detectable hydrolysis to a potentially mutagenic and carcinogenic primary aromatic amine.

## Discussion

To better understand the risk related to release of aromatic amides from food contact materials and potential transfer into food, the aim of the present work was to assess the stability of NDPA, NAAX and Naphthol AS and potential cleavage to primary aromatic amines during simulated passage through the gastrointestinal tract. To this end, we utilized an in vitro digestion protocol recommended by EFSA (2008) for the safety assessment of substances to be used in plastic food contact materials, as well as a static in vitro digestion model established by the National Institute for Public Health and the Environment (RIVM, Bilthoven, NL) (Oomen et al. 2003; Versantvoort et al. 2005). This model involves the sequential incubation of the test compound with saliva, gastric and intestinal juice simulants, and more closely simulates the chemical composition of digestive fluids, pH and residence time in each compartment as compared to the EFSA (2008) protocol.

Based on the model of Versantvoort et al. (2005), time-dependent hydrolysis of NDPA and NAAX resulting in formation of the primary aromatic amine 2,4-DMA was observed. Cleavage of NDPA occurred predominantly in gastric juice, whereas all digestive fluid simulants, i.e., saliva, gastric juice and intestinal juice, contributed to hydrolysis of NAAX to 2,4-DMA. The maximum extent of cleavage observed during simulated passage through the gastrointestinal tract was 0.022% for NDPA and 0.033% for NAAX. Aniline formation was not detected in any incubations of Naphthol AS with digestive fluid simulants.

Experiments performed using gastric fluid simulants according to EFSA Note for Guidance for Food Contact Materials (EFSA 2008) were in line with these findings, although higher rates of hydrolysis of NDPA (0.21%) and NAAX (0.053%) were recorded following 4 h incubation with 0.07 M HCl as gastric-juice simulant, possibly due to the lower pH as compared to that achieved by sequential incubation with saliva and gastric-juice simulant based on the somewhat more physiological model of Versantvoort et al. (2005). Thus, the slightly harsher conditions and longer incubation times recommended by EFSA (2008) may be considered a worst-case scenario of what might be expected to occur under physiological conditions in vivo. Despite these stringent conditions, there was no evidence for cleavage of Naphthol AS to aniline. Overall, these data are consistent with acid amides and acid anilides being generally very stable compounds (Carey and Sundberg 2000; O'Connor 1970; Warren et al. 2001). Experimental details of hydrolysis reactions of compounds structurally related to NDPA (Morar et al. 2015; Murai et al. 2016), NAAX (Sultana et al. 2018) and Naphthol AS (Singh and Jana 2016; Wang et al. 2016) reported in the literature show that hydrolysis of the amide bond requires vigorous conditions such as heat and strong acids or bases. Considering the chemical stability of acid amides, it should also be emphasized that omission of mucin and lipase, which were not commercially available at the time, from the digestive fluid simulants based on Versantvoort et al. (2005) is not expected to significantly affect cleavage rates. As high-molecular-weight glycosylated proteins, it is extremely unlikely that mucins would contribute to hydrolysis of acid amides. Likewise, there is no evidence from literature that lipases, which are specialized to catalyze the hydrolysis of triglycerides into free fatty acids and glycerol, play a role in xenobiotic metabolism.

Considering the lack of evidence for aniline formation from Naphthol AS and the extremely low rate of hydrolysis of the amide bonds of NDPA and NAAX during simulated passage through the gastrointestinal tract that gives rise to only very minor amounts of the potentially mutagenic and/or carcinogenic aromatic amine 2,4-DMA, the approach to risk assessment by the German Federal Institute for Risk Assessment (BfR) which assumed 100% cleavage to the primary aromatic amines would appear to significantly overestimate the health risks related to the presence of aromatic amides in food contact materials.

## Supplementary Information

Below is the link to the electronic supplementary material.

Supplementary file1 (DOCX 529 KB)

## Abbreviations

2,4-DMA: 2,4-Dimethylaniline
NAAX: *N*-Acetoacetyl-m-xylidine
NDPA: *N*-(2,4-Dimethylphenyl)acetamide

## Footnotes

1 Full report is available upon request from Walter@vci.de.
