# Supplementary material for: Assessment of aromatic amides in printed food contact materials: analysis of potential cleavage to primary aromatic amines during simulated passage through the gastrointestinal tract
Source: Arch Toxicol. 2022 Mar 5;96(5):1423–35. doi: 10.1007/s00204-022-03254-w (PMC9013685; doi:10.1007/s00204-022-03254-w)
Supplement: Supplementary file 1 — Supplementary file1 (DOCX 529 KB) [file 204_2022_3254_MOESM1_ESM.docx]

**Supplemental Data**

**Supplementary Figure 1**

**
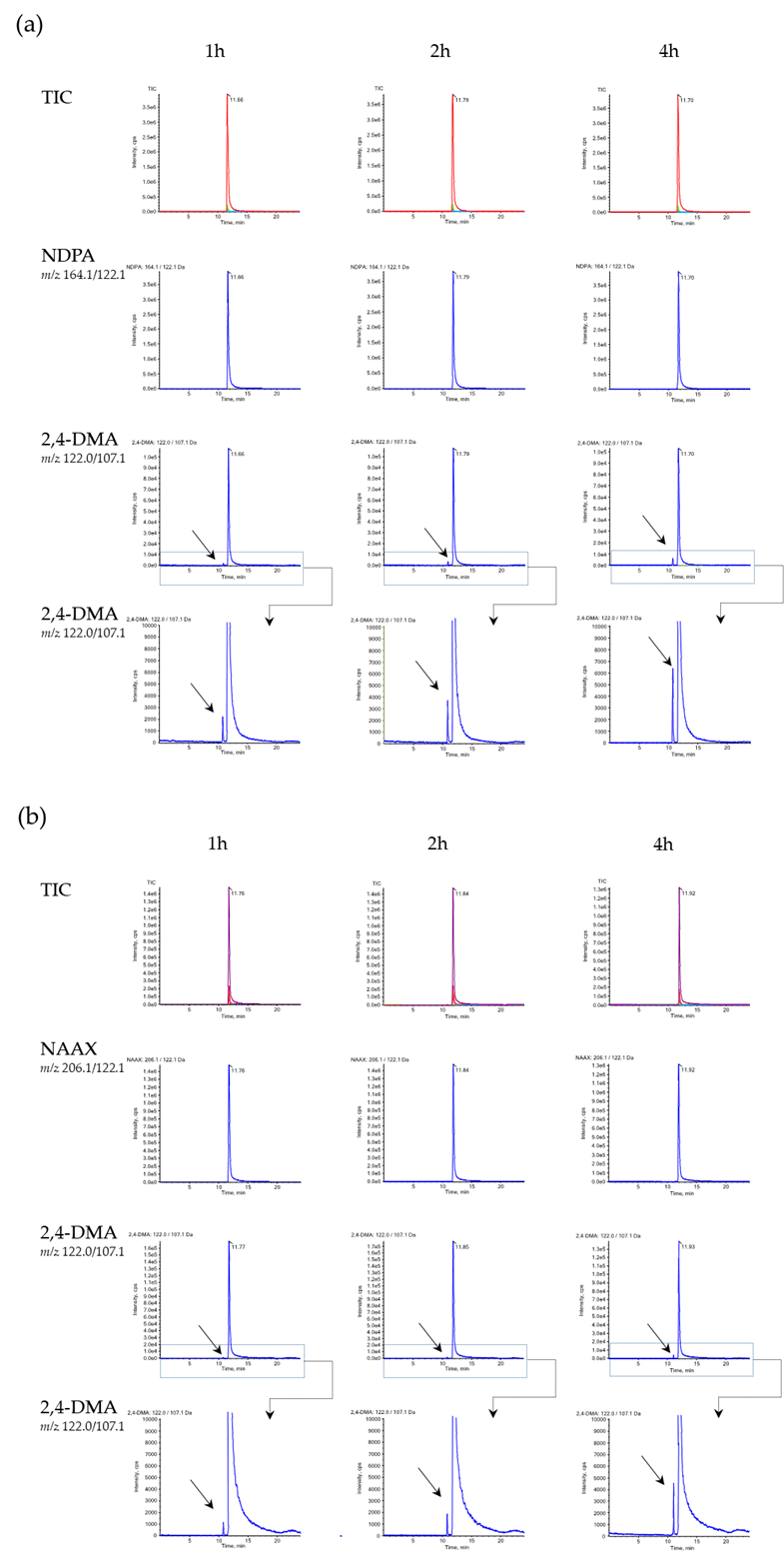
**

*Supplementary Figure 1 HPLC-ESI-MS/MS analysis demonstrating hydrolysis of NDPA (a) and NAAX (b) and formation of 2,4-DMA after incubation with gastric juice simulant (0.07M HCl) for 1, 2 and 4hrs. The signal of 2,4-DMA formed by cleavage of NDPA and NAAX is marked by an arrow, the larger signals with m/z 122.0/107.1 eluting at the same retention times as NDPA and NAAX, respectively, result from degradation of NDPA and NAAX to 2,4-DMA by electrospray.*

**Supplementary Figure 2**

**
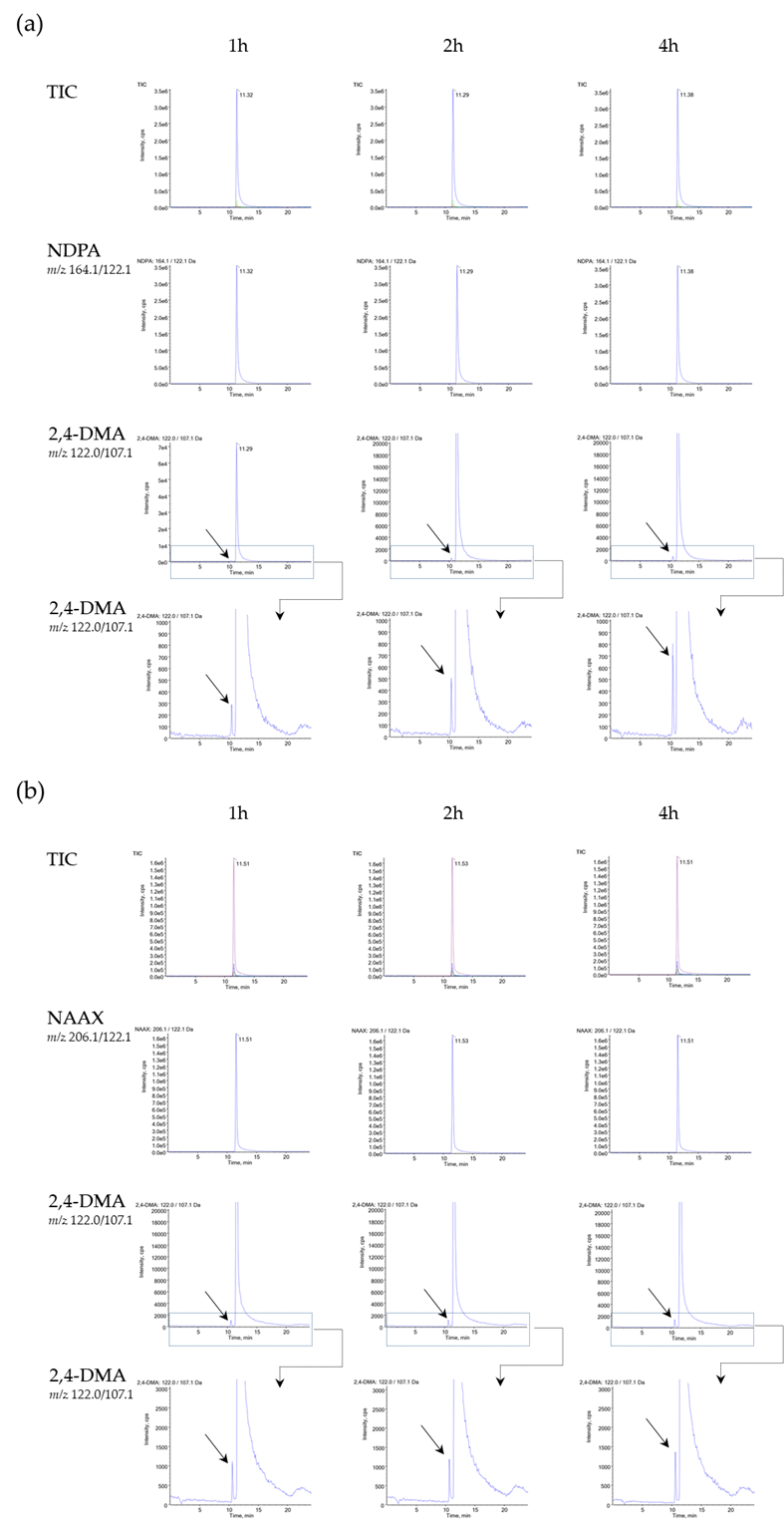
**

*Supplementary Figure 2 HPLC-ESI-MS/MS analysis of in vitro digestion of NDPA (a) and NAAX (b) after sequential incubation with saliva (0.5h) and gastrointestinal juice for 1, 2 and 4hrs, demonstrating time-dependent cleavage of NDPA and NAAX to 2,4-DMA. The signal of 2,4-DMA formed by cleavage of NDPA and NAAX is marked by an arrow, the larger signal with m/z 122.0/107.1 eluting at the same retention times as NDPA and NAAX, respectively, results from degradation of NDPA and NAAX to 2,4-DMA by electrospray.*

**Supplementary Figure 3**


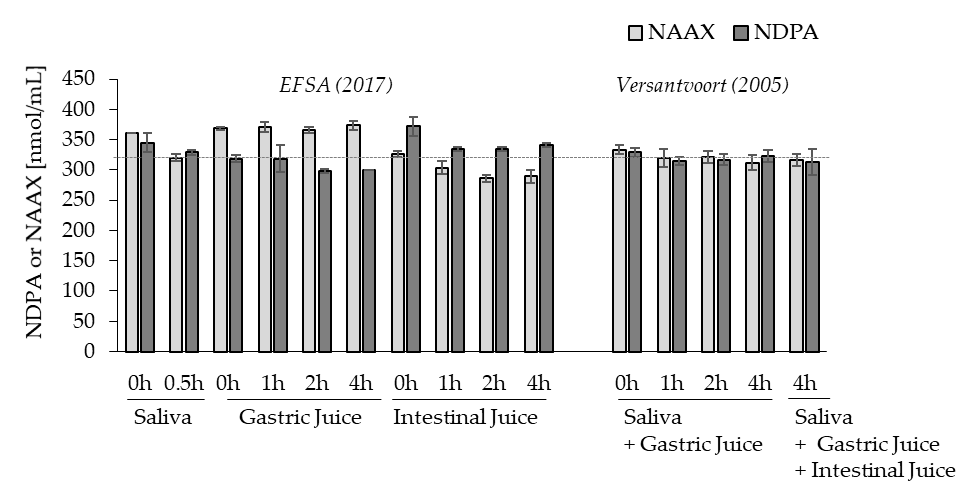


*Supplementary Figure 3 Quantitative analysis of NDPA and NAAX to 2,4-DMA following in vitro digestion based on the protocol of EFSA (2017) and Versantvoort et al. (2005). The final concentration of test compound was 318 nmol/mL in each incubation mixture. Data are presented as mean ± standard deviation (n=3). To account for reduced recovery in digestive fluid simulants, additional samples (t = 0h) were generated by addition of test compounds to the respective matrix and immediate injection into the LC-MS system.*

**Supplementary Figure 4**


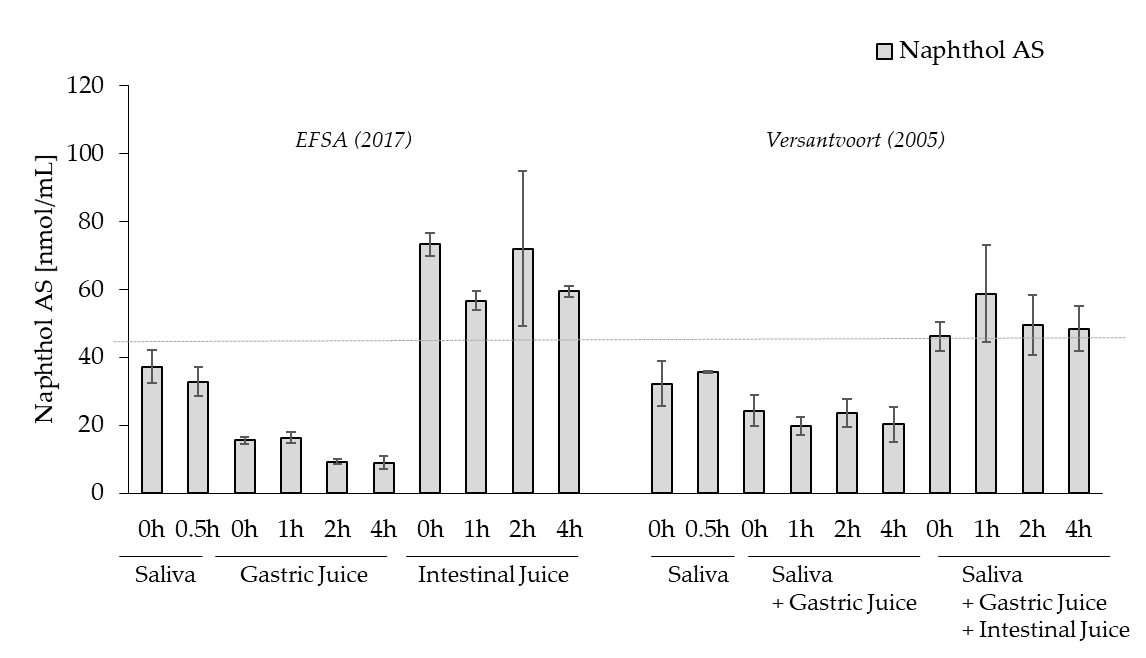


*Supplementary Figure 4 Quantitative analysis of Naphthol AS following incubation with digestive fluid simulants based on the protocol of EFSA (2017) and Versantvoort et al. (2005). Data are presented as mean ± standard deviation (n=3). The final concentration of test compound in each incubation mixture was 43.7 nmol/mL. To account for reduced recovery in digestive fluid simulants, additional samples (t = 0h) were generated by addition of Naphthol AS to the respective matrix and immediate injection into the LC-MS system.*
